# Managing Scarcity: Innovation and Resilience During the COVID-19 Pandemic

**DOI:** 10.3389/fsurg.2021.769962

**Published:** 2021-10-14

**Authors:** Natalie Pozzi, Aaron Zuckerman, Joohee Son, Travis C. Geraci, Stephanie H. Chang, Robert J. Cerfolio

**Affiliations:** ^1^Department of Cardiothoracic Surgery, NYU Langone Health, New York, NY, United States; ^2^Department of General Surgery, NYU Langone Health—Brooklyn, Brooklyn, NY, United States

**Keywords:** COVID, COVID-19 management, innovation, tracheostomy, bronchoscopy, telehealth, training

## Abstract

The Coronavirus Disease 2019 (COVID-19) pandemic remains a disruptive force upon the health care system, with particular import for thoracic surgery given the pulmonary pathophysiology and disease implications of the virus. The rapid and severe onset of disease required expedient innovation and change in patient management and novel approaches to care delivery and nimbleness of workforce. In this review, we detail our approaches to patients with COVID-19, including those that required surgical intervention, our expedited and novel approach to bronchoscopy and tracheostomy, and our expansion of telehealth. The pandemic has created a unique opportunity to reflect on our delivery of care in thoracic surgery and apply lessons learned during this time to “rethink” how to optimize resources and deliver excellent and cutting-edge patient care.

## Introduction

The accelerated pace of severe acute respiratory syndrome coronavirus 2 (SARS-CoV-2) and the consequent Coronavirus Disease 2019 (COVID-19) requires an equally rapid rate of change and flexibility in the management of critically ill patients. With particular focus on respiratory failure and thoracic complications, patients with COVID-19 challenges our current practices and inspired new approaches to care and treatment techniques. Patients presented with unique and oftentimes severe disease, while other patients posed the alternative challenge of avoiding in-personal evaluation and deferred routine care.

While vaccinations have been critical at stanching the pandemic, poorly informed citizens, and uninitiated workforce coupled with variant strains continue to push the capacity of hospitals and healthcare workers. During this unprecedented time, new methods of healthcare delivery such as telemedicine have been developed, allowing us unique opportunities to reflect on our standard delivery of care in thoracic surgery and to apply lessons learned during the pandemic to “rethink” how we optimize resources and deliver excellent patient care.

## Surgery for Patients With COVID-19

Patients with severe COVID-19 disease that suffer acute respiratory distress syndrome (ARDS) are at higher risk for developing pulmonary complications, including barotrauma in those who required mechanical ventilation, pneumatocele, and/or empyema from secondary bacterial infection, hemothorax in the setting of anticoagulation, or prolonged air-leak from a pleural-alveolar fistula. Given the risk of disease transmission, the health-care community was appropriately hesitant to perform thoracic surgical procedures, given the potential exposure during intubation, potential aerosolization during insufflation for minimally-invasive surgery, and the theoretical risk of exposure from air-leaks.

At our institution, the rate of hospitalized patients with COVID-19 who required thoracic surgery was low. Over a 5-month period, 1,899 patients were admitted with a positive rtPCR test and only 13 patients (0.7%) underwent a thoracic surgical intervention. Patients were taken for surgery for unresolving pneumothorax, pneumatocele with continued air leak, empyema, and hemothorax. Both minimally invasive and open techniques were performed. Overall these patients were critically ill with prolonged COVID-19 courses, with the median day of surgery on hospital day 43 (1).

Seven patients (54%) of the 13 who underwent an operation had unresolving air leaks. Prior to the procedure these patients were evaluated with a chest computed tomography scanning. Five patients (38%) were found to have one or more pneumatoceles. The majority of these procedures (86%) were performed with a minimally invasive technique either video-assisted thoracoscopic surgery (VATS) (57%) or robotic-assisted surgery (29%). One procedure (14%) was a thoracotomy. Lysis of adhesions were performed as necessary, wedge resections for findings of blebs or necrotic lung tissue follow by decortication and placement of multiple chest drains. Pneumatoceles were managed by either unroofing or resection followed by decortication. The thoracotomy was performed for a patient with a large loculated pneumothorax with bilateral pneumatoceles with hemodynamic instability.

Five patients (38%) were taken to the operating room for hemothorax in the setting of therapeutic anticoagulation. Patients were on therapeutic anticoagulation for known high rates of pulmonary embolism and thromboembolic complications in COVID-19 patients or for veno-venous extracorporeal membrane oxygenation (VV-ECMO) when requiring further respiratory support (2–4). Three patients (60%) underwent a VATS hemothorax evacuation and decortication and two patients (40%) had a thoracotomy secondary to ongoing hemorrhage. As before, any areas of necrosis were removed by wedge resection or lobectomy if required. One patient required a right lower lobectomy for infarction secondary to inferior pulmonary vein thrombosis (5). One patient (8%) underwent a robotic decortication in the setting of severe sepsis from Klebsiella pneumonia complicated by empyema. This patient also had resection of a necrotic area found in the right lower lobe.

The outcomes for these procedures were favorable with nine patients (69%) discharged from the hospital on room air. Three patients (23%) died postoperatively and one patient (8%) remained hospitalized. Deaths were related to ongoing respiratory failure in two patients and multisystem organ failure in one patient (1).

To reduce the risk of transmission, a number of practices were instated. Operating room procedures were exclusively performed in negative pressure rooms. Members of the operating team who did not scrub wore a hair cover, N95 mask, eye protection, non-sterile gown, and gloves throughout the entirety of the procedure. For scrubbed staff members, standard intraoperative sterile techniques were followed with the addition of N95 masks. During intubation, non-anesthesia personnel waited outside of the operating room. Postoperatively, patients were admitted to dedicated COVID-19 intensive care units. Hospital personnel involved in operative procedures were monitored closely for the development of symptoms with daily screening and monthly rtPCR testing. No surgeon or operating room staff contracted COVID-19 from a workplace exposure when caring for patients with COVID-19 in the operating room.

## Lung Cancer Management During the COVID-19 Pandemic

Lung cancer management during the COVID-19 pandemic was directed by local transmission rates and hospital capacity. A number of institutions changed their protocols, often delaying surgery or choosing alternative methods of treatment during the midst of the pandemic. At our institution, we chose to maintain our protocols of care for patients with lung cancer and performed surgery as needed while implementing certain safety measures.

During a non-surge phase with adequate hospital capacity, adjustments were made to preserve low levels of transmission and protect patients and healthcare personnel. Standard protocols were followed for lung cancer work-up and management. Patients were contacted by phone and screened for symptoms prior to any in-person visit. Healthcare providers wore N95 masks, eye shields, and gloves for all patient contact. Patients and visitors wore face coverings regardless of their symptoms (6).

Normal preoperative pulmonary function testing evaluation, bronchoscopy with biopsy or endobronchial ultrasound (EBUS) for staging was continued with appropriate precautions. Patients required negative COVID-19 testing within 3 days of the procedure and all staff for procedures wore N95 masks, eye shields, and gloves. When these precautions are taken there has been shown to be minimal infectious risk to provider with practically no transmission during bronchoscopy or EBUS (7, 8).

During surge phases of high local transmission and limited hospital capacity more conservative measures were taken for lung cancer management to reduce hospital procedures and minimize the risk of postoperative infectious complications. Staging endobronchial ultrasounds were routinely deferred in newly-diagnosed patients without radiographic lymphadenopathy, especially with low standardized uptake value (SUV) of the primary lesion on PET CT. Conversely, patients with imaging findings highly convincing for advanced or metastatic disease were managed without tissue confirmation. Patients with planned radiation therapy were wither treated with delivery of a hypo-fractionated regiment or single fraction delivery. Though all cases were still discussed at multi-disciplinary thoracic oncology conference (MDTOC) prior to making changes to standard treatment plans.

Patients necessitating intervention, such as those with symptomatic central airway obstruction, suspected recurrent lung cancer, or malignant pleural effusions underwent bronchoscopy, diagnostic, and therapeutic procedures as needed under appropriate precautions for patients and providers. The patients had to have negative COVID-19 tests within 3 days of the procedure and providers wore the previously mentioned PPE. For malignant pleural effusions, indwelling pleural catheter (IPC) insertion was preferred over VATS and tac pleurodesis since it could be performed on an outpatient basis and a family member trained in the care and drainage of the IPC.

Operative procedures for curative resection were planned based on the guidelines set forth by the *American College of Surgeons* and the *Thoracic Surgery Outcomes Research Network* and discussion at our institutional MDTOC. Patients with a solid or >50% solid nodule >2 cm, SUVmax >2.5, or change on short interval CT scan were more likely to have an impact on survivorship with surgical delay. On the other hand, surgery was delayed for ~3 months for patients with predominantly ground glass nodules, solid nodules <2 cm, or radiographic findings of an indolent tumor (9).

With these criteria applied during the initial peak of the COVID-19 pandemic in New York City and a surge of cases and hospitalizations from March to May 2020, our hospital had 21 patients undergo surgical resection. There were no major complications and an average hospital length of stay of 2 days. None of these patients developed symptoms or tested positive for COVID-19. Of the patients who chose to defer surgery (24 patients), only one patient showed progression from T3N0 to T4N0 disease. There were nine healthcare providers involved in the surgical procedures for these patients and none of them contracted COVID-19 (10).

In a review from high volume centers throughout the world, Seitlinger et al. report the safety and feasibility of operations for malignant thoracic diseases during the COVID-19 pandemic (11). In a total of 306 open procedures and 428 minimally-invasive procedures, there were no statistically different outcomes of patients undergoing surgery before or during the pandemic for lung cancer. Given the risk of progression of disease and therefore a higher stage at time of definitive intervention, lung cancer management should not be delayed. Our own experience and data from other centers have shown this can be accomplished while preserving outcomes and protecting health care workers.

Furthermore, during the pandemic, we accelerated our goal of sending patients home on postoperative day number one after pulmonary resection—a practice we had integrated into our care system prior to the pandemic. With the theory that patients would recover optimally at home after surgery—avoiding iatrogenesis, reducing the risks of delirium, and particular to the pandemic, avoiding infection—we sought to discharge all patients on postoperative day number one. With the risk of contracting COVID-19 in the hospital, thereby risking pulmonary complications in the postoperative period, patients were equally motivated for enhanced recovery. In a series of 253 patients, 134 (53%) were discharged by postoperative day number one (12). Hospital discharge was influenced by patient baseline status, complications, and extent of resection. In 12% of cases, patients with an air-leak were discharged with a chest tube and managed as an outpatient. In the setting of a real potential for contacting an infectious disease while hospitalized, expedited hospital discharge has been proved safe while maintaining excellent postoperative outcomes.

This is critically important, as data reporting on patients who develop COVID-19 after pulmonary resection have worse outcomes. In a series from Italy, Scarci et al., reported a 50% mortality rate in patients who developed COVID-19 after pulmonary resection within 90 days (HR 4.49, 95% CI: 2.71–5.82, *P* < 0.001). Additionally, body mass index, smoking status, and number of lung segments resected were associated with the risk of developing COVID-19 in the postoperative period. These outcomes highlight the importance of preventing COVID-19 transmission in the postoperative period after lung resection. Limiting hospitalization and utilizing telehealth (discussed below) are two key methods for reducing this risk.

## Tracheostomy and Bronchoscopy for COVID-19 Respiratory Failure

Severe COVID-19 infection can lead to rapid and prolonged respiratory failure in susceptible patients. Bronchoscopy is often needed in this population to clear mucus plugs or to obtain bronchoalveolar lavage specimens, and many patients with ongoing respiratory failure require tracheostomy to prevent complications from orotracheal intubation. Since the clinical course of this patient population was not known early in the course of the pandemic, the safety to patients of these procedures was questioned. Furthermore, given the uncertain nature of SARS-CoV-2 transmission early on, it was uncertain if these procedures would be safe to providers, or if aerosolized particles would transmit the virus from patients to healthcare workers. Major societies in otolaryngology and thoracic surgery in March of 2020 adamantly rejected performing tracheostomies on these patients for fear of infecting all the bedside healthcare provides. We believe this disserved the patient and questioned these policies and mandates since they did not seem evidence based. We saw patients asphyxiate from tenacious secretions and believed early tracheostomy would help. Therefore, we innovated and developed a safe and unique method for tracheostomy.

To mitigate the risk of transmission of SARS-CoV-2 to providers, we described an approach to percutaneous dilational tracheostomy (PDT) in which the bronchoscope was passed alongside the endotracheal tube rather than inside it (13). In 96 patients with favorable ventilator settings (positive end-expiratory pressure of 12 mmHg or less, fraction of inspired oxygen 60% or less, respiratory rate 25 or fewer breaths per minute, and partial pressure of carbon dioxide 60 mmHg or less) and without multiorgan failure, PDT was performed. In the 1-month data collection period, no major complications or mortality was attributed to our method of PDT and 33% of patients who underwent PDT no longer required ventilatory support. None of the eight providers performing the tracheostomies had any symptoms of COVID-19 infection or tested positive for the virus following the procedures. A later propensity score matched analysis of 205 patients at our institution who underwent tracheostomy (195 via our novel PDT protocol) compared patients who underwent early tracheostomy (median = 9 days of MV), late tracheostomy (median = 19 days of MV), or no tracheostomy (14). Compared to patients who did not require tracheostomy, early PDT was associated with more days of MV but higher probability of being liberated from the ventilator and lower mortality. Compared to late PDT, early PDT was associated with fewer ventilator days, higher rate of discontinuing MV, and no difference in mortality. Furthermore, photometric analysis during the procedures did not demonstrate increased aerosolization from baseline MV, and again, no spread of SARS-CoV-2 was noted to healthcare providers performing these procedures. Our data suggests the safety and potential benefit in early PDT, in contrast to most domestic guidelines (15). As more studies are performed, the ideal timing of PDT may become clear.

In our experience with COVID-19 patients, bronchoscopy can be performed in the intensive care setting safely with respect to both patients and providers. In a retrospective analysis of mechanically ventilated patients with COVID-19 at our institution, 241 bronchoscopies were performed on 107 patients (7). All bronchoscopies were performed in a negative pressure room with providers wearing full personal protective equipment. Patients were preoxygenated before starting, and the procedures were carried out under neuromuscular blockade with the ventilator disconnected. Patients were reconnected to the ventilator if oxygen saturations dropped below 90%. No patient suffered any major adverse event attributed to bronchoscopy using this technique, and of the nine proceduralists who did not test positive for COVID-19 beforehand, none had a positive test within 2 weeks after performing a bronchoscopy. Furthermore, bronchoscopy in this population resulted in higher positive culture rate than tracheal aspiration (65 vs. 45%), and 6% of bronchoalveolar lavage samples grew different or additional organisms compared to tracheal aspirates. Similar results were found in a retrospective analysis by Bruyneel et al., who additionally document the safety and efficacy of a cleaning protocol by which disposable bronchoscopic equipment could be used multiple times for the same patient (16). In their study, 90 bronchoscopies were performed on 32 patients in the intensive care unit. COVID-19 patients in their intensive care unit requiring bronchoscopy had a non-significantly higher unadjusted mortality rate than similar patients who did not require bronchoscopy. While further studies are warranted, current evidence supports the safety and utility of bronchoscopy in COVID-19 intensive care patients.

## Telemedicine and Outpatient Care During the COVID-19 Pandemic

In the COVID-19 pandemic, medical practices across all disciplines transformed their practices to limit in-person contact and expand their telemedicine services. Even though telemedicine has been utilized previously, the COVID-19 pandemic exponentially increased the resources and coverage available for telemedicine. The US Center for Medicare and Medicaid Services (CMS), for example, expanded its coverage for telemedicine visits, adding to the development and use of telemedicine platforms (17). Grenda et al. described their hospital system's transition to telemedicine services during the COVID-19 pandemic (18). In one visit, they were able to incorporate multiple disciplines for those with lung cancer, and provide a seamless approach in all aspects of pre- and post-operative care.

In a prospective study done at our institution, 56 patients underwent telemedicine visits for their pre- and post-operative care. In the post-operative period, most patients (33) had only telemedicine visits, and six patients were able to avoid an emergency room visit. Satisfaction surveys were sent, and 96% of the patients gave the surgeon the highest score in all areas of patient communication (19). This study showed that telemedicine can maintain high integrity in communication and safety and increase patient satisfaction by eliminating a potentially long trip, especially if patients live far away from the institution.

Overall, there are many benefits to the utilization of telemedicine in thoracic surgery. Through telemedicine, hospitals can optimize resources to deliver quality and necessary care to patients while maintaining social distancing. Additionally, visits with multiple providers can be scheduled for one telemedicine visit, especially for oncology patients, making it a seamless process. One of the biggest limitations in telemedicine is ensuring access to care for all patients, as some patients may not have access or be able to afford the technology that is required for telemedicine. Continued research and expansion of care for all patients is ongoing to ensure that all patients have the necessary care they need.

## Surgical Training During the Pandemic

The COVID-19 pandemic not only disrupted the health care system regarding patient care delivery, but also challenged the initiation of clinical training for new health care providers and medical students and modified or delayed the ongoing education of residents and fellows. For thoracic surgery, assuring that trainees are able to obtain and maintain technical proficiency during the pandemic invited novel approaches to learning surgery. In a survey of cardiothoracic trainees, over half reported a case-volume reduction of over 50% (20). Equally, trainees reported losing time in the hospital and/or having clinical duties changed to manage COVID-19 related work. In another survey from cardiothoracic trainees in the United Kingdom, Caruana et al. found that 63 and 32% of respondents reported concerns about their physical and mental health due to the COVID-19 pandemic. Furthermore, there was a significant impact on time spent in clinics (44% reduction), multidisciplinary meetings (79% reduction), and operating time (78% reduction). The majority (88%) were concerned about the impact of the pandemic on their training and most of these (71%) felt that the deviation may require an extension in their planned training time.

While there remain many opportunities for learning during this seemingly unending pandemic, particularly in critical care, surgical skills and exposure, patient care was often curtailed for prolonged periods of time as case volumes shrank. In this setting, simulation and virtual learning became two strategies that developed with accelerated pace during the pandemic. For example, a virtual technical skills course was developed to teach the essential steps of lung transplantation when the equivalent operative experience was not available. As Chan et al. describe, the simulation experience, which was bolstered with biweekly video coaching sessions, improved the technical skills, and confidence in performing lung transplantation in the majority of trainees who underwent the program (21). Lessons learned from this experience, the authors conclude, can be used to create simulation curricula for trainees across a wide breadth of operations. At our institution, to help curtail potential deficits in training, we sought to continue weekly didactic learning sessions and multidisciplinary conferences via virtual platforms. Equally, we sought to maintain surgical simulation labs and promoted review of surgical videos to help master and maintain operative techniques. As Caruana et al. concludes, “the COVID-19 pandemic poses significant and professional challenges to trainees. Nonetheless, it is a teachable moment—focusing on core values, professionalism, quality, and safety of care.”

## Conclusion

Every dark storm, no matter the devastation or morbidity left behind, has a silver lining. Yet, only the initiated who not only laments the loss, but actively seeks the gains clearly view it. The COVID-19 pandemic, despite is lethality, is no exception. Leadership that invokes innovation and nimbleness both in command structure and implementation is the hallmark of the many amazing stories, outcomes and heroic saves that COVID-19 delivered. These qualities are part of a highly reliable organization. Yet these qualities only lead to outstanding performance and executive function when this culture is coupled with highly dedicated and highly trained people. It is the people that make it happen. People who work together.

We believe this concept of “optimizing resources and maximizing patient outcomes” often occurs when managing via scarcity. It is a blend of leadership, dedication, and resilient people. The true heroes on the ground is the entre workforce that was at the bedside or helped to delivered supplies to those at the bedside. People who rose and fought every day, day after day that made the difference. Stellar outcomes achieved at NYU Langone Health and throughout the world, were secondary to our culture and to the amazing people who gave of themselves and placed their lives and their families' lives at risk in order to serve out patients (22). Our patients are our most prized commodity and resource.

## Author Contributions

NP, AZ, JS, TG, SC, and RC contributed equally to the formation, writing, and editing of this manuscript. All authors contributed to the article and approved the submitted version.

## Conflict of Interest

RC discloses relationships with AstraZeneca, Bard Davol, Bovie Medical Corporation, C-SATS, ConMed, Covidien/Medtronic, Ethicon, Fruit Street Health, Google/Verb Surgical, Intuitive Surgical, KCI/Acelity, Myriad Genetics, Neomend, Pinnacle Biologics, ROLO-7, Tego, and TransEnterix. The remaining authors declare that the research was conducted in the absence of any commercial or financial relationships that could be construed as a potential conflict of interest.

## Publisher's Note

All claims expressed in this article are solely those of the authors and do not necessarily represent those of their affiliated organizations, or those of the publisher, the editors and the reviewers. Any product that may be evaluated in this article, or claim that may be made by its manufacturer, is not guaranteed or endorsed by the publisher.
